# The Experimental Infections of the Human Isolate of *Strongyloides Stercoralis* in a Rodent Model (The Mongolian Gerbil, *Meriones Unguiculatus*)

**DOI:** 10.3390/pathogens8010021

**Published:** 2019-02-05

**Authors:** Sarit Charuchaibovorn, Vivornpun Sanprasert, Surang Nuchprayoon

**Affiliations:** 1 Medical Sciences Program, Faculty of Medicine, Chulalongkorn University, Bangkok 10330, Thailand; sarit.src@gmail.com; 2 Lymphatic Filariasis and Tropical Medicine Research Unit, Chulalongkorn Medical Research Center, Faculty of Medicine, Chulalongkorn University, Bangkok 10330, Thailand; vivornpun@chula.md; 3 Department of Parasitology, Faculty of Medicine, Chulalongkorn University, Bangkok 10330, Thailand

**Keywords:** strongyloidiasis, *Strongyloides stercoralis*, Mongolian gerbil (*Meriones unguiculatus*), hyperinfection, dissemination

## Abstract

Strongyloidiasis is life-threatening disease which is mainly caused by *Strongyloides stercoralis* infection. Autoinfection of the parasite results in long-lasting infection and fatal conditions, hyperinfection and dissemination (primarily in immunosuppressed hosts). However, mechanisms of autoinfection and biology remain largely unknown. Rodent models including mice and rats are not susceptible to the human isolate of *S. stercoralis*. Variations in susceptibility of the human isolate of *S. stercoralis* are found in dogs. *S. ratti* and *S. venezuelensis* infections in rats are an alternative model without the ability to cause autoinfection. The absence of appropriate model for the human isolate of strongyloidiasis hampers a better understanding of human strongyloidiasis. We demonstrated the maintenance of the human isolate of the *S. stercoralis* life cycle in the Mongolian gerbil (*Meriones unguiculatus*). The human isolate of *S. stercoralis* caused a patent infection in immunosuppressed gerbils, more than 18 months. The mean number of recovery adult parasitic worms were 120 ± 23 (1.2% of the initial dose) and L1s were 12,500 ± 7500 after day 28 post-inoculation (p.i.). The prepatent period was 9–14 days. Mild diarrhoea was found in gerbils carrying a high number of adult parasitic worms. Our findings provided a promising model for studying biology and searching new alternative drugs against the parasites. Further studies about the hyperinfection and dissemination would be performed.

## 1. Introduction

Strongyloidiasis is a parasitic disease, caused by *Strongyloides* spp. infections. *Strongyloides stercoralis* is a major pathogenic species which is capable of infecting humans, dogs and non-human primates. *S. stercoralis* infection causes significant health problems throughout the world, particularly in Sub-Saharan Africa, South-America and Southeast Asia [1,2]. Approximately, 370 million people are infected with the parasite [3,4,5]. The most unique characteristic of the parasite is the ability to multiply and complete their life cycle within hosts as a result of so-called “autoinfection.” In immunocompetent hosts, *S. stercoralis* can cause a long-lasting infection, up to 75 years [6,7]. In immunocompromised hosts, the cycle of autoinfection can dramatically increase resulting in potentially life-threatening conditions, including hyperinfection and dissemination. The autoinfective larvae (L3a) can penetrate through the intestinal wall into other organs and cause death due to organ failure and sepsis. Mortality rates of the hyperinfection and dissemination syndrome are 34.6–87% [8,9,10,11]. Moreover, fatal hyperinfection cases are also found, even in immunocompetent hosts [12,13]. The mortality rate of hyperinfection in immunocompetent hosts is 22% [13]. The mechanisms of autoinfection and biology of *S. stercoralis* are largely unknown. To obtain a better understanding of *S. stercoralis* infection in a human, an appropriate animal model is required.

In the 1980s, dogs and Patas monkeys were used as a model for strongyloidiasis studies [14,15,16,17,18]. However, they are unsatisfactory to be a laboratory model, due to a few disadvantages, such as difficulty of handling and high maintenance costs. Moreover, there are variabilities in susceptibility of dogs to the human isolate of *S. stercoralis* [14,19,20,21]. Cats and ferrets are also demonstrated for *S. stercoralis* infection, however, they are not able to develop a chronic infection as found in humans. Most rodent models, including mice, rats, guinea pigs are not susceptible to *S. stercoralis* [22]. Although SCID mice is susceptible to *S. stercoralis*, only 0.02–0.3% of infective larvae can develop into adult parasitic worms, even in a high initial dose of infective larvae [23]. Although *S. ratti* and *S. venezuelensis* infections in rats are used as an alternative model for strongyloidiasis study, these parasites lack the ability to cause the autoinfection which is the unique feature of *S. stercoralis* infection in humans [24,25,26]. The Mongolian gerbil (*Meriones unguiculatus*) is an important model for strongyloidiasis study. Gerbils are able to develop a chronic infection, up to 131 days. Moreover, complicated conditions (hyperinfection and dissemination) are developed in immunosuppressed animals [27]. However, most of the recent studies of the gerbil model used the dog isolate *S. stercoralis* [28,29,30], while human isolate *S. stercoralis* is less developed in gerbils. Although marmoset is able to develop uncomplicated and complicated strongyloidiasis, using human isolate *S. stercoralis,* they are less than ideal laboratory host [31].

In this study, we developed the gerbil model for the human isolate of *S. stercoralis*. The human isolate of *S. stercoralis* developed a chronic infection, more than 18 months, in immunosuppressed gerbils. The findings of this study provide a useful model for the study of biology and novel drug research of strongyloidiasis.

## 2. Results

### 2.1. Immunosuppressed Gerbils Were Susceptible to the Human Isolate of S. stercoralis

**Figure 1:**
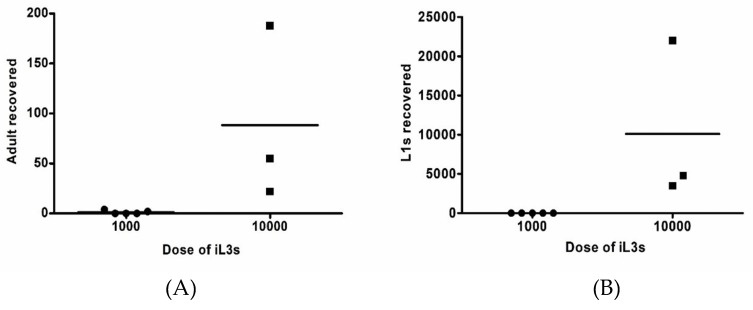
Recovery of *S. stercoralis* from gerbils infected with the human isolate of *S. stercoralis* (obtained from human faecal cultures). The number of adult parasitic worms (**A**) and L1s (**B**) were represented.

Thirteen Mongolian gerbils were immunosuppressed with 2 mg methylprednisolone (MPA) and inoculated by subcutaneous injection with 1000 (group 1, N = 7) or 10,000 (group 2, N = 6) of *S. stercoralis* infective stage larvae (iL3s) that were obtained from human faecal cultures (Table 1). The susceptibility of infections was observed daily by faecal direct smear and agar plate after day 7 post-inoculation (p.i.). In group 1, two of five male gerbils were positive for *S. stercoralis* infection by agar plates after day 9 p.i, while two female gerbils died unexpectedly on day 3 p.i. (Table 1). The direct smear was negative in all gerbils (Table 1). After day 28 p.i., a few adult parasitic worms (2–4 worms) were recovered from intestines of the two male gerbils (Table 1 and Figure 1A). No L1s and L3a were found from the infected animals. With higher inoculation dose (group 2), all 4 male gerbils were positive for *S. stercoralis* on direct smear and agar plates after day 9 p.i., while the 2 female gerbils were negative. After day 28 p.i., three infected gerbils were euthanized, while the remaining gerbil (no. 11) was used as the source of iL3s for the later experiment (group 3–8) and still alive at 18 months. Adult parasitic worms were found in small and large intestines. The number of recovered adult parasitic worms from infected male gerbils varies from 22 to 188 worms and L1s were between 3500 and 22,000 worms (Table 1 and Figure 1). A few L1s were found in the lungs of one gerbil (gerbil no. 8). No L3a were recovered from infected animals. Mild diarrhoea was noted in infected gerbils carrying a high number of adult parasitic worms (gerbil no. 8). 

### 2.2. The Human Isolate of S. stercoralis Life Cycle Could Be Maintained by Gerbil-to-Gerbil Transfer

**Figure 2:**
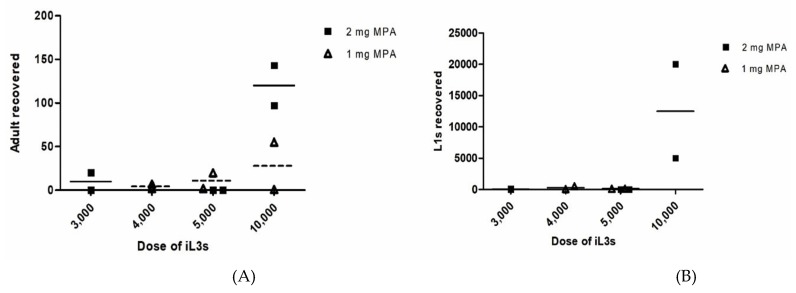
Recovery of *S. stercoralis* from gerbils infected with the human isolate of *S. stercoralis* (obtained from gerbil faecal cultures) (1 mg of MPA versus 2 mg of MPA). The number of adult parasitic worms (**A**) and L1s (**B**) were represented.

To maintain *S. stercoralis* life cycle by gerbil-to-gerbil transfer, we suspected that gerbils were more susceptible to human isolate *S. stercoralis* which were obtained from infected gerbils obtained from strongyloidiasis patient. We decreased doses of MPA and varied initial doses of iL3s. Six gerbils were immunosuppressed with 1 mg MPA and two each inoculated with 4000 (group 3), 5000 (group 4) or 10,000 (group 5) *S. stercoralis* iL3s that were obtained from gerbil No.11 faecal cultures. All gerbils faeces were positive for *S. stercoralis* by agar plates, while group 3 and 4 were negative by direct smear after day 9 p.i. (Table 1). The number of recovered adult parasitic worms varies from 1 to 55 without relationship with inoculation dosage (Table 1 and Figure 2A). Similarly, the number of recovered L1s varies from 66 to 500 without relationship with inoculation dosage (Table 1 and Figure 2B). Unfortunately, the number of L1s in gerbil group 5 could not be counted or estimated, due to the presence of numerous of the stool faecal debris in intestines. No L3a were found from infected animals. 

The experiment was repeated with higher degree of immunosuppression. Nine gerbils were immunosuppressed with 2 mg MPA and two each were inoculated with 3000 (group 6), 5000 (group 7) and 5 gerbils with 10,000 (group 8) *S. stercoralis* iL3s that were obtained from gerbil No.11 faecal cultures. One gerbil in group 6 and three gerbils in group 8 were positive for *S. stercoralis* by direct smear and agar plates after day 9 p.i., while rest was negative (Table 1). Two female gerbils in group 8 were also positive by agar plates. Two gerbils in group 7 died unexpectedly on day 3 and 7 p.i. One gerbil from the lowest inoculation (group 6) did not get infected. After day 28 p.i., the infected gerbil from group 6, 2 male and 2 female gerbils from group 8 were euthanized. The remaining male gerbil in group 8 still alive for 8 months. The number of recovered adult parasitic worms varies from 2 to 143 without relationship with inoculation dosage (Table 1 and Figure 2A). The number of recovered L1s varies from 110 to 20,000 without relationship with inoculation dosage (Table 1 and Figure 2B). A few L1s were found in the lungs of a gerbil in group 8 (gerbil no. 26). A few L3a were recovered in the lungs (gerbil no. 26), representing an autoinfection. 

## 3. Discussion

The absence of appropriate laboratory model for the human isolate of strongyloidiasis hampers a better understanding of *S. stercoralis* infection in humans. The previous studies showed that the Mongolian gerbil is susceptible to the dog isolate of *S. stercoralis* [32,33,34]. However, the dog isolate of *S. stercoralis* might be different from the human isolate of *S. stercoralis.* Here, we demonstrate that the immunosuppressed gerbils is a suitable laboratory animal to maintain the life cycle of the human isolate of *S. stercoralis*. 

**Figure 3:**
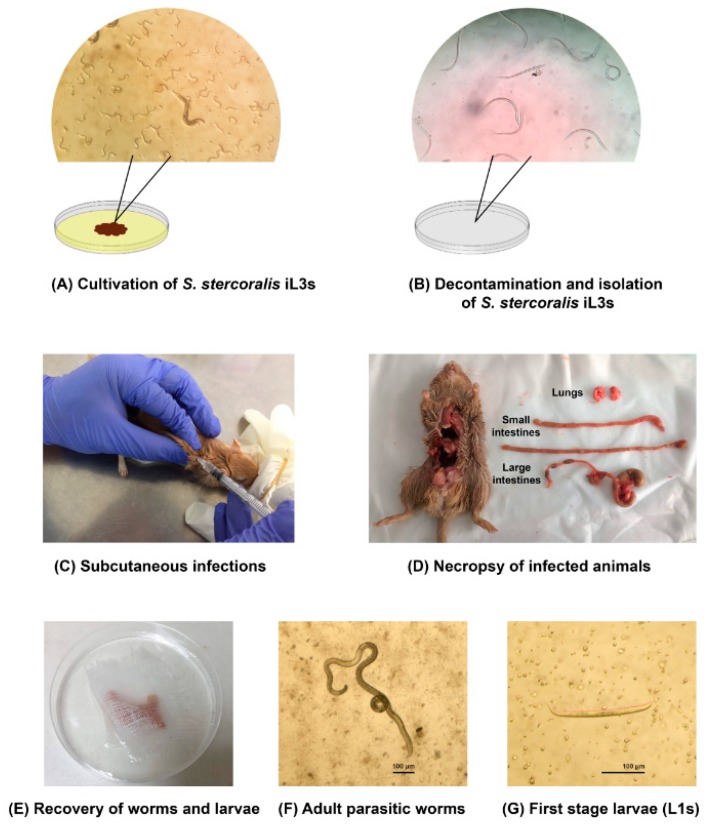
The process of experimental infections of human isolate of *S. stercoralis* in gerbils.

Although linear relationship between the inoculation dosage and adult worm recovery from gerbil is not consistent, higher inoculation dosage of iL3s and MPA result in a high yield of worms and larvae from gerbils (Figure 2 and Figure 3). Unfortunately, the differences were not statistically significant. It might be due to small size of animals per group. We found that male gerbils were more susceptible to *S. stercoralis* infection more than female gerbils, (Table 1). This observation is consistent with the results using the dog isolate of *S. stercoralis.* [27] Moreover, the source of iL3s (obtained from strongyloidiasis patients and infected gerbils) did not influence the number of recovered adult parasitic worms in gerbils.

Our percentage of recovery adult parasitic worms (1.2%) were slightly higher than recovery worms in immunocompetent gerbils (0.29%) [34]. The percentage of recovered adult parasitic worms of the human isolate of *S. stercoralis* were lower than the using the dog isolate of *S. stercoralis* (7%) [27,34] The recovered L1s in this study was higher than the number of recovery worms in immunocompetent gerbils up to ~ 20 folds [34]. The fecundity (L1s/adult ratio) was ~ 104.1, while the fecundity of the dog isolate of *S. stercoralis* was 4.0–21.7 [27]. The result of coproparasitological examination by agar plates showed that the L1s, excreted in faeces, were active and numerous, consistent with the number of larvae recovered from the gut.

The human isolate of *S. stercoralis* can cause a persistent infection in MPA-treated gerbils as found in humans (*S. stercoralis,* obtained from strongyloidiasis patients: for at least 18 months and *S. stercoralis,* obtained from infected gerbils: for at least 8 months). The prepatent period was 9–14 days. Mild diarrhoea (common clinical sign of human strongyloidiasis) was found in infected gerbils carrying a high number of adult parasitic worms (>100 worms/gerbil). Focal lesions were also presented at the larval or MPA injection sites of a high number of adult parasitic worms, as previously found in a previous study [32]. Other clinical symptoms were not found. 

The most important characteristic of human strongyloidiasis is that the disease can be fatal in immunocompromised hosts, due to hyperinfection and dissemination. The two fatal conditions arose from increasing of autoinfection rates in which the L1s in the host’s intestines become the L3a and complete their parasitic life cycle in the host, especially in immunocompromised hosts. However, the small number of L3a were found in 2 mg MPA-treated gerbils, even L1s were numerous in the intestines. The results were not consistent with the results from using dog isolate of *S. stercoralis* [27,34]. Either the characteristics of the parasites or the animal hosts affected the susceptibility. We suspected that the discrepancies might be due to several factors. The autoinfection of dog isolate of *S. stercoralis* in gerbils was induced by two factors, including treatment with MPA and injection with a high dose of iL3s [27,34]. 

The corticosteroid drug is a major trigger for the development of autoinfection [8,9,35]. The minimum dose of MPA to induce the autoinfection of the dog isolate of *S. stercoralis* in gerbils was 1 mg (Few L3a were found.). However, the number of L3a dramatically increased 400 fold when increased to a dose of 2 mg MPA [27]. It is probably that using the higher dose of MPA (more than 2 mg) may induce autoinfection of human isolate of *S. stercoralis* in gerbils.

The genetic factors might influence the susceptibility of gerbils to the human isolate of *S. stercoralis*. There were variabilities in the susceptibility of the human isolate of *S. stercoralis* in dogs [14,19,20,21,36]. The human isolate of *S. stercoralis* is usually incapable to develop a patent infection in dogs. The genetic analysis between humans and dog isolate of *S. stercoralis* showed the variations in Cox1, 18S rDNA and 28S rDNA genes [37,38,39,40,41,42]. Further studies about the genetics of humans and dog isolate of *S. stercoralis* could be performed to get a better understanding about the susceptibility to gerbils. Moreover, a different isolate of *S. stercoralis* might also influence the efficiency of infection. There was a variability of *S. ratti* infection in rats. The usual dose to initiate an infection in the rat is 500 iL3s, however, there was a severe case of using 500 iL3s of *S. ratti* wild isolate in the rat [26]. 

In summary, we demonstrated the use of immunosuppressed gerbils as a laboratory model to maintain the human isolate of *S. stercoralis* life cycle. In this study, we showed that the human isolate of *S. stercoralis* is able to develop a patent infection in MPA-treated gerbils, more than 18 months, mimicking the chronicity of human strongyloidiasis. Interestingly, a number of recovered L1s from this study were comparable to recovered L1s from gerbils infected with the dog isolate *S. stercoralis* when the autoinfection occurred [27,34], even though the number of recovered adult parasitic worms from this study were lower than using the dog isolate of *S. stercoralis* [27,34]. Further studies aim to induce the autoinfection in gerbils infected with the human isolate of *S. stercoralis* by increasing doses of MPA and/or initial doses of iL3s. Moreover, the histopathology of the human isolate of *S. stercoralis* would be demonstrated. Our findings provided a promising model for studying biology and searching new alternative drugs against the parasites. Using gerbils has advantages in ease of handling and breeding and cost.

## 4. Materials and Methods 

### 4.1. Animals

The Mongolian gerbils (*Meriones unguiculatus*) were purchased from the Zooya farm (Bangkok, Thailand) at about 4 weeks of age. The animals were screened negative for bacterial-, viral and parasitic infections prior to entering into the facility. They were kept 2–4 animals/cage under controlled air and temperature at 25 ± 2 °C. Gerbils were bred together in captivity for two generations. The F2 generation was used in experimental infections. The animals were provided with food and water *ad libitum*. All animal experimental protocols were carried out under the approval by the Chulalongkorn University Animal Care and Use Committee, Faculty of Medicine, Chulalongkorn University, Bangkok, Thailand (CU-ACUC approval no: 022/2560).

### 4.2. Immunosuppression of Animals

To induce the autoinfection in gerbils, 6–12 weeks old gerbils were subcutaneously injected with 200 μL of 10–20 mg/mL MPA (total 1–2 mg, depending on the experiments) at day −3, 0, 7, 14 and 21 p.i. 

### 4.3. The Human Isolate of S. stercoralis Infective Stage (iL3s)

The human isolate of *S. stercoralis* was harvested from faecal specimens of strongyloidiasis patients from the King Chulalongkorn Memorial Hospital, Bangkok, Thailand. Agar plate cultures were set up to obtain iL3s for the experimental infections [43]. Approximately, 1–3 grams of stool specimens were placed on the centre of agar plates and incubated at 25 °C for 5 days (Figure 3A). The iL3s were collected from the surface of positive agar plates by washing with the buffer saline solution (BU saline) (22 mM KH_2_PO_4_, 50 mM Na_2_HPO_4_, 70 mM NaCl). The iL3s were decontaminated by using low melting point agarose and then axenized in BU saline with 100 U/mL penicillin, 10 µg/mL streptomycin and 12.5 µg/mL tetracycline for 3 h (Figure 3B). 

### 4.4. Experimental Infections

Immunosuppressed gerbils (6–12 weeks old) were subcutaneously injected with a suspension of 1000–10,000 iL3s (depending on the experiment) in 200 μL of inoculation volume on day 0 p.i. (Figure 3C). Each gerbil was separated for 1 animal/cage under controlled air and temperature 25 °C ± 2. There were 8 groups of experimental infections (Table 1). Group 1 and 2 aimed to test whether immunosuppressed gerbils were susceptible to the human isolate of *S. stercoralis,* gerbils in group 1 and 2 were immunosuppressed with 2 mg MPA and injected with 1000 and 10,000 of *S. stercoralis* iL3s (obtained from human faecal cultures), respectively. Group 3–8 aimed to test whether *S. stercoralis* could be maintained by gerbil-to-gerbil transfer, gerbil no. 11 from group 2 were used as a source of iL3s for experimental infections in group 3–8. Gerbils in group 3–5 were immunosuppressed with 1 mg MPA and injected with 4000, 5000 and 10,000 of *S. stercoralis* iL3s (obtained from gerbil faecal cultures), respectively. Gerbils in group 6-8 were immunosuppressed with 2 mg MPA and injected with 3000, 5000 and 10,000 of *S. stercoralis* iL3s (obtained from gerbil faecal cultures), respectively. *S. stercoralis* infection in gerbils was confirmed by the presence of larvae in stool and parasitic adult worms in the intestines.

### 4.5. Coproparasitological Examination

To determine the susceptibility of infections and observe the prepatent period of the infections, a semi-quantitative estimation of the number of L1s from infected animals were performed. Approximately, 2 grams of faecal specimens were collected and cultured daily by using agar plates, after day 7 p.i. The agar plates were observed daily for 5 days under a stereo microscope. The faecal culture results were categorized by convention as: – (No tracks of migration and/or larvae were found.); + (Only tracks of migration were found.); ++ (Free-living adults were first seen on day 4–5.); +++ (Numerous free-living adults were first seen on day 3.).

### 4.6. Necropsy and Recovery of S. stercoralis

On day 28 p.i., gerbils were anesthetized and then euthanized by cervical dislocation. The intestines and lungs of infected animals were slit longitudinally (Figure 3D) and individually wrapped with gauze (Figure 3E). The organs were soaked in BU saline and incubated at 37 °C for 3 h. After incubation, the contents of the intestines and lungs were examined for the presence of adult worms and larvae under a stereo microscope. The morphology of adult parasitic worms and L1s were represented in Figure 3F,G, respectively.

### 4.7. Statistics Analysis

All data were represented in the text and figures as a mean ± standard error. Averages between groups were compared and tested for statistical significance by using the unpaired *t*-test. A *P* value of < 0.05 was considered statistically significant.

## Figures and Tables

**Table 1:** Recovery of *S. stercoralis* adult parasitic worms and L1s 28 days after initial infection of gerbils with *S. stercoralis* iL3s.

Source of iL3s	Group	No.	Gender	Dose of iL3s	Dose of MPA	Direct Smear	Agar Plate	Recovered Adult Parasitic Worms	Recovered L1s
Human	1	1	Male	1000	2 mg	−	+	4	NF
		2				−	+	2	NF
		3				−	−	NF	NF
		4				−	−	NF	NF
		5				−	−	NF	NF
		6	Female	1000	2 mg	Death			
		7				Death			
	2	8	Male	10,000	2 mg	+	+++	188	22,000
		9				+	++	22	4800
		10				+	++	55	3500
		11				+	+++	ND *	ND *
		12	Female	10,000	2 mg	−	−	NF	NF
		13				−	−	NF	NF
Gerbil	3	14	Male	4000	1 mg	−	+	7	66
		15				−	+	2	500
	4	16	Male	5000	1 mg	−	+	20	110
		17				−	+	2	140
	5	18	Male	10,000	1 mg	+	++	1	ND **
		19				+	++	55	ND **
	6	20	Male	3000	2 mg	+	++	20	110
		21				−	−	NF	NF
	7	22	Male	5000	2 mg	Death			
		23				Death			
	8	24	Male	10,000	2 mg	+	+++	143	20,000
		25				+	+++	97	5000
		26				+	+++	ND *	ND *
		27	Female	10,000	2 mg	−	++	2	NF
		28				−	++	44	2000

Note: −: No tracks of migration and/or larvae were found; +: Only tracks of migration were found; ++: Free-living adults were first seen on day 4-5; +++: Numerous free-living adults were first seen on day 3; *: Animals still alive; **: The number of L1s could not be counted and estimated, due to a presence of numerous of stool faecal debris in intestines; NF: not found; ND: no data.

## References

[1] Krolewiecki A.J., Lammie P., Jacobson J., Gabrielli A.F., Levecke B., Socias E., Arias L.M., Sosa N., Abraham D., Cimino R. (2013). A public health response against Strongyloides stercoralis: Time to look at soil-transmitted helminthiasis in full. PLoS Negl. Trop. Dis..

[2] Schar F., Trostdorf U., Giardina F., Khieu V., Muth S., Marti H., Vounatsou P., Odermatt P. (2013). Strongyloides stercoralis: Global Distribution and Risk Factors. PLoS Negl. Trop. Dis..

[3] Bisoffi Z., Buonfrate D., Montresor A., Requena-Mendez A., Munoz J., Krolewiecki A.J., Gotuzzo E., Mena M.A., Chiodini P.L., Anselmi M. (2013). Strongyloides stercoralis: A plea for action. PLoS Negl. Trop. Dis..

[4] Farthin M., Albonico M., Bisoffi Z., Buonfrate D., Katelaris P., Kelly P., Savioli L., Le Mair A. Management of Strongyloidiasis. http://www.webcitation.org/75si8tJzH.

[5] Beknazarova M., Whiley H., Judd J.A., Shield J., Page W., Miller A., Whittaker M., Ross K. (2018). Argument for Inclusion of Strongyloidiasis in the Australian National Notifiable Disease List. Trop. Med. Infect. Dis..

[6] Gill G.V., Beeching N.J., Khoo S., Bailey J.W., Partridge S., Blundell J.W., Luksza A.R. (2004). A British Second World War veteran with disseminated strongyloidiasis. Trans. R. Soc. Trop. Med. Hyg..

[7] Prendki V., Fenaux P., Durand R., Thellier M., Bouchaud O. (2011). Strongyloidiasis in man 75 years after initial exposure. Emerg. Infect. Dis..

[8] Marcos L.A., Terashima A., Canales M., Gotuzzo E. (2011). Update on strongyloidiasis in the immunocompromised host. Curr. Infect. Dis. Rep..

[9] Buonfrate D., Requena-Mendez A., Angheben A., Munoz J., Gobbi F., Van Den Ende J., Bisoffi Z. (2013). Severe strongyloidiasis: A systematic review of case reports. BMC Infect. Dis..

[10] Kim J.H., Kim D.S., Yoon Y.K., Sohn J.W., Kim M.J. (2016). Donor-Derived Strongyloidiasis Infection in Solid Organ Transplant Recipients: A Review and Pooled Analysis. Transplant. Proc..

[11] Vazquez Guillamet L.J., Saul Z., Miljkovich G., Vilchez G.A., Mendonca N., Gourineni V., Lillo N., Pinto M., Baig A., Gangcuangco L.M. (2017). Strongyloides Stercoralis Infection Among Human Immunodeficiency Virus (HIV)-Infected Patients in the United States of America: A Case Report and Review of Literature. Am. J. Case Rep..

[12] Myint A., Chapman C., Almira-Suarez I., Mehta N. (2017). Strongyloides hyperinfection syndrome in an immunocompetent host resulting in bandemia and death. BMJ Case Rep..

[13] Chan F.L.Y., Kennedy B., Nelson R. (2018). Fatal Strongyloides hyperinfection syndrome in an immunocompetent adult with review of the literature. Intern. Med. J..

[14] Grove D.I., Northern C. (1982). Infection and immunity in dogs infected with a human strain of Strongyloides stercoralis. Trans. R. Soc. Trop. Med. Hyg..

[15] Genta R.M., Harper J.S., Gam A.A., London W.I., Neva F.A. (1984). Experimental disseminated strongyloidiasis in Erythrocebus patas. II. Immunology. Am. J. Trop. Med. Hyg..

[16] Harper J.S., Genta R.M., Gam A., London W.T., Neva F.A. (1984). Experimental disseminated strongyloidiasis in Erythrocebus patas. I. Pathology. Am. J. Trop. Med. Hyg..

[17] Schad G.A., Hellman M.E., Muncey D.W. (1984). Strongyloides stercoralis: Hyperinfection in immunosuppressed dogs. Exp. Parasitol..

[18] Barrett K.E., Neva F.A., Gam A.A., Cicmanec J., London W.T., Phillips J.M., Metcalfe D.D. (1988). The immune response to nematode parasites: Modulation of mast cell numbers and function during Strongyloides stercoralis infections in nonhuman primates. Am. J. Trop. Med. Hyg..

[19] Sandground J.H. (1928). Some studies on susceptibility, resistance, and acquired immunity to infection with Strongyloides stercoralis (Nematoda) in dogs and cats. Am. J. Epidemiol..

[20] Augustine D.L., Davey D.G. (1939). Observations on a natural infection with Strongyloides in the dog. J. Parasitol..

[21] Galliard H. (1967). Pathogenesis of Strongyloides. Helminthol. Abstr..

[22] Dawkins H.J., Grove D.I. (1982). Attempts to establish infections with Strongyloides stercoralis in mice and other laboratory animals. J. Helminthol..

[23] Rotman H.L., Yutanawiboonchai W., Brigandi R.A., Leon O., Nolan T.J., Schad G.A., Abraham D. (1995). Strongyloides stercoralis: Complete life cycle in SCID mice. Exp. Parasitol..

[24] Lok J.B., Shao H., Massey H.C., Li X. (2017). Transgenesis in Strongyloides and related parasitic nematodes: Historical perspectives, current functional genomic applications and progress towards gene disruption and editing. Parasitology.

[25] Breloer M., Abraham D. (2017). Strongyloides infection in rodents: Immune response and immune regulation. Parasitology.

[26] Viney M., Kikuchi T. (2017). Strongyloides ratti and S. venezuelensis—Rodent models of Strongyloides infection. Parasitology.

[27] Nolan T.J., Megyeri Z., Bhopale V.M., Schad G.A. (1993). Strongyloides stercoralis: The first rodent model for uncomplicated and hyperinfective strongyloidiasis, the Mongolian gerbil (Meriones unguiculatus). J. Infect. Dis..

[28] Li X., Massey H.C., Nolan T.J., Schad G.A., Kraus K., Sundaram M., Lok J.B. (2006). Successful transgenesis of the parasitic nematode Strongyloides stercoralis requires endogenous non-coding control elements. Int. J. Parasitol..

[29] Junio A.B., Li X., Massey H.C., Nolan T.J., Todd Lamitina S., Sundaram M.V., Lok J.B. (2008). Strongyloides stercoralis: Cell- and tissue-specific transgene expression and co-transformation with vector constructs incorporating a common multifunctional 3′ UTR. Exp. Parasitol..

[30] Albarqi M.M., Stoltzfus J.D., Pilgrim A.A., Nolan T.J., Wang Z., Kliewer S.A., Mangelsdorf D.J., Lok J.B. (2016). Regulation of Life Cycle Checkpoints and Developmental Activation of Infective Larvae in Strongyloides stercoralis by Dafachronic Acid. PLoS Pathog..

[31] Mati V.L., Raso P., de Melo A.L. (2014). Strongyloides stercoralis infection in marmosets: Replication of complicated and uncomplicated human disease and parasite biology. Parasit Vectors.

[32] Kerlin R.L., Nolan T.J., Schad G.A. (1995). Strongyloides stercoralis: Histopathology of uncomplicated and hyperinfective strongyloidiasis in the Mongolian gerbil, a rodent model for human strongyloidiasis [corrected]. Int. J. Parasitol..

[33] Sithithaworn P., Fujimaki Y., Mitsui Y., Prasanthong R., Yutanawiboonchai W., Aoki Y. (1998). Efficacy of ivermectin against Strongyloides stercoralis infection in jirds (Meriones unguiculatus). Exp. Parasitol..

[34] Nolan T.J., Bhopale V.M., Rotman H.L., Abraham D., Schad G.A. (2002). Strongyloides stercoralis: High worm population density leads to autoinfection in the jird (Meriones unguiculatus). Exp. Parasitol..

[35] Croker C., Reporter R., Redelings M., Mascola L. (2010). Strongyloidiasis-related deaths in the United States, 1991–2006. Am. J. Trop. Med. Hyg..

[36] Genta R.M. (1989). Strongyloides stercoralis: Loss of ability to disseminate after repeated passage in laboratory beagles. Trans. R. Soc. Trop. Med. Hyg..

[37] Neefs J.M., Van de Peer Y., De Rijk P., Chapelle S., De Wachter R. (1993). Compilation of small ribosomal subunit RNA structures. Nucleic Acids Res..

[38] Ramachandran S., Gam A.A., Neva F.A. (1997). Molecular differences between several species of Strongyloides and comparison of selected isolates of S. stercoralis using a polymerase chain reaction-linked restriction fragment length polymorphism approach. Am. J. Trop. Med. Hyg..

[39] Hasegawa H., Hayashida S., Ikeda Y., Sato H. (2009). Hyper-variable regions in 18S rDNA of Strongyloides spp. as markers for species-specific diagnosis. Parasitol. Res..

[40] Hasegawa H., Sato H., Fujita S., Nguema P.P., Nobusue K., Miyagi K., Kooriyama T., Takenoshita Y., Noda S., Sato A. (2010). Molecular identification of the causative agent of human strongyloidiasis acquired in Tanzania: Dispersal and diversity of *Strongyloides* spp. and their hosts. Parasitol. Int..

[41] Schar F., Guo L., Streit A., Khieu V., Muth S., Marti H., Odermatt P. (2014). Strongyloides stercoralis genotypes in humans in Cambodia. Parasitol. Int..

[42] Nagayasu E., Aung M., Hortiwakul T., Hino A., Tanaka T., Higashiarakawa M., Olia A., Taniguchi T., Win S.M.T., Ohashi I. (2017). A possible origin population of pathogenic intestinal nematodes, Strongyloides stercoralis, unveiled by molecular phylogeny. Sci. Rep..

[43] Koga K., Kasuya S., Khamboonruang C., Sukhavat K., Ieda M., Takatsuka N., Kita K., Ohtomo H. (1991). A modified agar plate method for detection of Strongyloides stercoralis. Am. J. Trop. Med. Hyg..

